# Vitamin D levels and prolonged menstrual cycle in women with polycystic ovary syndrome: a cross-sectional study

**DOI:** 10.3389/fnut.2026.1785886

**Published:** 2026-05-13

**Authors:** Lijing Wang

**Affiliations:** Department of Reproductive Health and Infertility, Zhaoqing Maternal and Child Health Care Hospital, Zhaoqing, China

**Keywords:** fitted curve, inflection point analysis, PCOS, prolonged menstrual cycle, vitamin D

## Abstract

**Objective:**

This study evaluated serum vitamin D (VD) levels in women with polycystic ovary syndrome (PCOS) and assessed their association with menstrual cycle characteristics.

**Methods:**

In this cross-sectional study, 449 women diagnosed with PCOS were stratified based on 25-hydroxyvitamin D [25(OH)D] levels: low VD group (<20 ng/mL) and normal VD group (≥20 ng/mL). Menstrual cycles of 26–35 days were defined as normal; cycles exceeding 35 days were classified as prolonged. All participants underwent measurements of body mass index (BMI), fasting plasma glucose (FPG), fasting insulin (FINS), and sex hormones.

**Results:**

The prevalence of prolonged menstrual cycles was significantly higher in the low VD group (87.2%) compared to the normal VD group (70%) (*p* < 0.001). Regression analysis demonstrated an inverse association between VD levels and the risk of prolonged cycles (*p* < 0.001). This association remained significant after adjusting for age, BMI, HOMA-IR, and total testosterone. Each 1 ng/mL increase in VD was associated with an 9% reduction in the risk of prolonged cycles. Fitted curves indicated a non-linear relationship between VD levels and the prevalence of prolonged cycles. Inflection point analysis identified VD = 27.76 ng/mL (approximately 28 ng/mL) as the inflection point. Below this level, the prevalence of prolonged cycles decreased as VD increased; above 28 ng/mL, the prevalence plateaued.

**Conclusion:**

In this cross-sectional study, lower vitamin D levels were associated with a higher prevalence of prolonged menstrual cycles among women with PCOS. This association suggests that further investigation into whether maintaining vitamin D levels at or above 28 ng/mL could influence menstrual cycle regularity in this population is warranted.

## Introduction

Polycystic Ovary Syndrome (PCOS) is a prevalent endocrine and metabolic disorder affecting women of reproductive age, characterized by oligomenorrhea, hyperandrogenism (HA), and polycystic ovarian morphology (1). Its global prevalence ranges from 5 to 20% (2). Oligomenorrhea, defined as menstrual cycles exceeding 35 days or fewer than eight periods annually, constitutes a core diagnostic criterion and frequent clinical manifestation of PCOS (1, 2). This condition reflects dysregulation of the hypothalamic–pituitary-ovarian (HPO) axis and ovulatory dysfunction, and is associated with long-term health risks including infertility, endometrial hyperplasia, and potential carcinogenesis (3).

In recent years, the role of vitamin D (VD) in reproductive health has attracted increasing attention. Beyond its critical role in calcium-phosphorus metabolism and bone health, the active metabolite of vitamin D, 1,25-dihydroxyvitamin D3, functions as a steroid hormone. Binding to vitamin D receptors (VDRs)—distributed widely in tissues including the ovaries, endometrium, hypothalamus, and pituitary gland—it regulates follicular development, sex hormone synthesis, insulin sensitivity, and inflammatory responses (4, 5). Numerous studies report that PCOS patients frequently present with vitamin D deficiency or insufficiency, exhibiting markedly reduced serum 25-hydroxyvitamin D [25(OH)D] levels compared to age-matched healthy women (6, 7). Low vitamin D status in PCOS is associated not only with metabolic abnormalities such as insulin resistance, obesity, and metabolic syndrome (8, 9), but may also contribute to reproductive dysfunction pathogenesis by affecting HPO axis function and the ovarian microenvironment (5, 10).

Several cross-sectional and interventional studies suggest a potential association between low vitamin D levels and menstrual irregularity in PCOS patients (11, 12). For instance, research indicates that lower vitamin D levels correlate with more severe menstrual disturbances (13, 14). However, studies specifically examining the link between vitamin D status and defined menstrual phenotypes in PCOS, such as oligomenorrhea, remain limited, and findings are inconsistent. Crucially, most existing studies either employ dichotomous comparisons (deficiency: <20 ng/mL vs. sufficiency: ≥20 ng/mL) or rely on linear correlation analyses. These approaches fail to adequately explore potential non-linear dose–response relationships between vitamin D levels and oligomenorrhea risk, particularly overlooking the identification of inflection points or threshold effects.

Therefore, this cross-sectional study comprehensively investigates the relationship between serum 25(OH)D levels and oligomenorrhea risk in a PCOS cohort. Beyond comparing oligomenorrhea prevalence between low VD (<20 ng/mL) and normal VD (≥20 ng/mL) groups based on the common clinical threshold, we specifically employed statistical modeling to analyse the dose–response pattern and identify potential inflection points (thresholds). These findings will provide refined epidemiological evidence regarding vitamin D’s role in PCOS-related menstrual disturbances and may offer a rationale for future interventions targeting specific vitamin D thresholds to improve menstrual cyclicity.

## Materials and methods

### Study population

This cross-sectional study employed the hospital information system to consecutively retrieve medical records of patients with confirmed polycystic ovary syndrome (PCOS), who underwent serum 25-hydroxyvitamin D [25-(OH)D] testing at our reproductive medicine outpatient department between July 2022 and June 2025. Inclusion criteria: (1) age 18–44 years; (2) confirmed PCOS diagnosis; and (3) availability of complete vitamin D (VD) test data. Exclusion criteria: (1) history of vitamin D supplementation within 3 months prior to inclusion; (2) hepatic/renal disorders or malabsorption syndromes affecting VD metabolism; and (3) ongoing hormonal therapy or menstrual cycle-modulating treatments at the time of visit. PCOS diagnosis was based on the 2003 Rotterdam criteria, requiring the presence of at least two of the following features: (1) ovulatory dysfunction (oligomenorrhea or amenorrhea); (2) clinical hyperandrogenism (e.g., hirsutism, acne) or biochemical hyperandrogenemia; and (3) polycystic ovarian morphology on ultrasound (2). Diagnoses were documented after ruling out other conditions including congenital adrenal hyperplasia, Cushing’s syndrome, and androgen-secreting tumors. Ethics approval was obtained from the Ethics Committee of Zhaoqing Maternal and Child Health Hospital (Approval No. 20251204001). Given the retrospective design, the requirement for written informed consent was waived.

### Methods

During outpatient consultations, demographic characteristics, medical history, and menstrual patterns were recorded for all participants. Standardized physical examinations included anthropometric measurements (height, body weight, waist circumference, hip circumference) and transvaginal ultrasonography for antral follicle counting. Polycystic ovarian morphology (PCOM) was defined as either ≥12 antral follicles (2–9 mm diameter) per ovary or ovarian volume >10 mL (15). Participants were stratified according to serum 25-hydroxyvitamin D [25(OH)D] concentrations: <20 ng/mL (low VD group) or ≥20 ng/mL (normal VD group) (16). Menstrual cycles of 26–35 days were classified as normal, while those exceeding 35 days were defined as prolonged menstrual cycles (including oligomenorrhea and amenorrhea) (17).

Fasting venous blood samples were drawn on days 2–5 of the menstrual cycle or during random visits for amenorrhoeic participants. Participants fasted for 8–12 h prior to sampling, consuming only water. Serum 25-hydroxyvitamin D [25(OH)D] concentrations were measured using a Maglumi 4,000 automated chemiluminescence analyzer (Shenzhen New Industries Biomedical Engineering Co., Ltd., Shenzhen, China), serum concentrations of follicle-stimulating hormone (FSH), luteinising hormone (LH), estradiol (E2), total testosterone (TT), progesterone (P4), insulin, glucose, homocysteine (HCY) and thyroid-stimulating hormone (TSH) were measured using a Maglumi 6,000 automated chemiluminescence analyzer (Shenzhen New Industries Biomedical Engineering Co., Ltd., Shenzhen, China), while androstenedione (A4) and sex hormone-binding globulin (SHBG) levels were quantified with a Cosmic automated chemiluminescence immunoassay system (Cosmic Biotechnology Co., Ltd., Chongqing, China).

### Statistical analysis

Continuous variables are expressed as mean ± standard deviation or median (interquartile range) [M (IQR)], according to data distribution. Categorical variables are described as frequencies (percentages) [n (%)]. Intergroup comparisons were conducted using: independent samples *t*-tests for normally distributed continuous variables, Mann–Whitney *U* tests for non-normally distributed continuous variables, Chi-square (χ^2^) tests for categorical variables. Binary logistic regression models assessed associations between vitamin D status and prolonged menstrual cycles. An unadjusted model (Model 1) was initially developed, followed by a multivariable model adjusted for age and BMI (Model 2) and adjusted for age, BMI, HOMA-IR, and TT (Model 3). Results are reported as odds ratios (OR) with 95% confidence intervals (95% CI). Covariates were selected based on prior literature (13, 18, 19). To minimize bias and preserve the uncertainty associated with missing data, multiple imputation was performed (20). Five complete datasets were created using the fully conditional specification (FCS) method. Predictive mean matching (PMM) was used for continuous variables, and binary logistic regression was applied to binary categorical variables. Restricted cubic splines (RCS) with three knots evaluated potential non-linear dose–response relationships between serum vitamin D concentrations and menstrual cycle prolongation risk, by applying the fully adjusted model for age, BMI, HOMA-IR, and TT. When non-linearity was detected (*p* < 0.05), piecewise logistic regression models identified threshold effects and inflection points. All analyses were performed using R software (v4.2.2) and the Free Statistics platform (v2.1). Statistical significance was defined as two-tailed *p* < 0.05.

## Results

### Baseline characteristics stratified by vitamin D status

This study included 449 women with PCOS. Demographic and clinical characteristics were summarized in Table 1, with missing data for variables presented in Supplementary Table S1. Serum 25-hydroxyvitamin D [25(OH)D] concentrations ranged from 6.83 to 44.91 ng/mL (mean: 22.17 ng/mL). Participants were categorized as low VD (<20 ng/mL, *n* = 179) or normal VD (≥20 ng/mL, *n* = 270). Participants with low VD were slightly younger than those with normal VD (29.12 ± 4.17 vs. 29.99 ± 4.25 years; *p* = 0.033). No significant differences were observed in BMI, fasting glucose, fasting insulin, HOMA-IR, TT, TSH, HCY, or reproductive hormones (LH, FSH, LH/FSH ratio, A4, SHBG, E2, P4, PRL) between groups. The prevalence of prolonged menstrual cycles was markedly higher in the low VD group (87.15% vs. 70.00%, *p* < 0.001). Conversely, a higher proportion of women in the normal VD group exhibited regular menstrual cycles (30.00% vs. 12.85%).

**Table 1:** Baseline characteristics of the study population.

Variables	Total (*n* = 449)	Low VD group (*n* = 179)	Normal VD group (*n* = 270)	*p*
Age (y)	29.65 ± 4.24	29.12 ± 4.17	29.99 ± 4.25	0.033
BMI (kg/m^2^)	23.24 ± 4.29	23.56 ± 4.77	23.02 ± 3.93	0.194
TSH (uIU/ml)	2.19 ± 1.28	2.16 ± 1.22	2.20 ± 1.32	0.757
HCY (umol/l)	8.38 ± 2.58	8.37 ± 1.65	8.38 ± 3.04	0.974
FPG (mmol/l)	5.41 ± 0.63	5.43 ± 0.67	5.39 ± 0.60	0.493
FINS (mU/l)	16.18 ± 9.97	17.22 ± 11.13	15.50 ± 9.09	0.090
HOMA-IR	3.97 ± 2.61	4.24 ± 2.90	3.79 ± 2.38	0.090
A4 (nmol/l)	5.53 ± 2.18	5.70 ± 2.31	5.41 ± 2.08	0.224
SHBG (nmol/l)	33.94 (22.70, 52.73)	35.76 (21.08, 53.81)	33.03 (23.44, 51.84)	0.935
FSH (IU/L)	7.21 ± 1.73	7.18 ± 1.95	7.23 ± 1.58	0.813
LH (IU/L)	8.94 ± 5.80	8.80 ± 5.94	9.03 ± 5.71	0.715
TT (ng/ml)	0.42 ± 0.24	0.44 ± 0.24	0.41 ± 0.24	0.285
E2 (pg/ml)	44.54 ± 29.09	44.20 ± 30.16	44.75 ± 28.45	0.861
P4 (ng/ml)	0.40 (0.30, 0.52)	0.39 (0.30, 0.51)	0.40 (0.30, 0.52)	0.753
PRL (ng/ml)	20.24 (13.37, 28.07)	20.33 (13.68, 28.36)	19.96 (12.96, 28.07)	0.565
LH/FSH	1.28 ± 0.82	1.27 ± 0.84	1.28 ± 0.82	0.941
Menstrual cycle, *n* (%)				<0.001
Normal	104 (23.16)	23 (12.85)	81 (30.00)	
Prolonged menstrual cycle	345 (76.84)	156 (87.15)	189 (70.00)	

Data are presented as mean ± standard deviation (SD) for continuous variables with normal distribution or as median (IQR) for continuous variables that did not show a normal distribution, and categorical variables are reported as no. (%). BMI, body mass index; TSH, thyroid stimulating hormone; HCY, homocysteine; FPG, fasting plasma glucose; FINS, fasting insulin; HOMA-IR, Homeostatic Model Assessment of Insulin Resistance; A4, androstenedione; SHBG, sex hormone binding globulin; FSH, follicle stimulating hormone; LH, luteinizing hormone; E2, estradiol; TT, total testosterone; P4, progesterone; PRL, prolactin.

### Association between vitamin D status and prolonged menstrual cycle risk

To investigate the association between serum 25(OH)D concentrations and prolonged menstrual cycle risk, this study developed a series of logistic regression models based on existing empirical evidence and dataset characteristics. Given the elevated missingness rate of SHBG (Supplementary Table S1), TT was implemented as a surrogate covariate in primary models. Multicollinearity was rigorously assessed across all models using variance inflation factor (VIF) diagnostics (threshold VIF < 5.0; Supplementary Table S2), confirming the absence of significant collinear dependencies.

In the unadjusted Model 1 (Table 2), lower 25(OH)D concentrations were significantly associated with increased risk of prolonged menstrual cycles (OR = 0.90, 95% CI: 0.87–0.94; *p* < 0.001), suggesting a 10% risk reduction per 1 ng/mL increment in 25(OH)D. After adjusting for age and BMI in Model 2 (Table 2), this inverse association remained significant (OR = 0.91, 95% CI: 0.87–0.94; *p* < 0.001). Further adjustment for age, BMI, HOMA-IR, and TT in Model 3 (Table 2) demonstrated persistence of the inverse correlation (OR = 0.91, 95% CI: 0.87–0.94; *p* < 0.001), corresponding to a 9% decrease in prolonged cycle risk per 1 ng/mL increase in 25(OH)D.

**Table 2:** Logistic multivariate analysis of vitamin D levels and prolonged menstrual cycle.

Model No.	Variable	OR (95%CI)	*P*	Model Nagelkerke *R*^2^	Model significance *P*
Model 1	VD	0.90 (0.87 ~ 0.94)	<0.001	0.110	<0.001
Model 2	VD	0.91 (0.87 ~ 0.94)	<0.001		
	Age	1.00 (0.95 ~ 1.05)	0.857	0.114	<0.001
	BMI	1.05 (0.99 ~ 1.11)	0.078		
Model 3	VD	0.91 (0.87 ~ 0.94)	<0.001		
	Age	1.00 (0.95 ~ 1.05)	0.857		
	BMI	1.05 (0.99 ~ 1.11)	0.078	0.129	<0.001
	HOMA	1.21 (1.07 ~ 1.36)	0.002		
	TT	2.13 (0.71 ~ 6.42)	0.177		

Model 1: crude model.

Model 2: adjusted for age + BMI.

Model 3: adjusted for age+BMI+HOMA-IR+TT.

Given significant SHBG data absence, the free androgen index (FAI) was excluded from primary covariates. However, supplementary analyses implementing FAI as a surrogate for TT yielded congruent core association results (Supplementary Table S3). Furthermore, to validate alternative missing data handling approaches, a complete-case analysis (employing listwise deletion) was separately conducted (Supplementary Table S4), demonstrating concordance in key effect estimates with the primary analysis.

### Non-linear association and inflection point analysis

**Figure 1:**
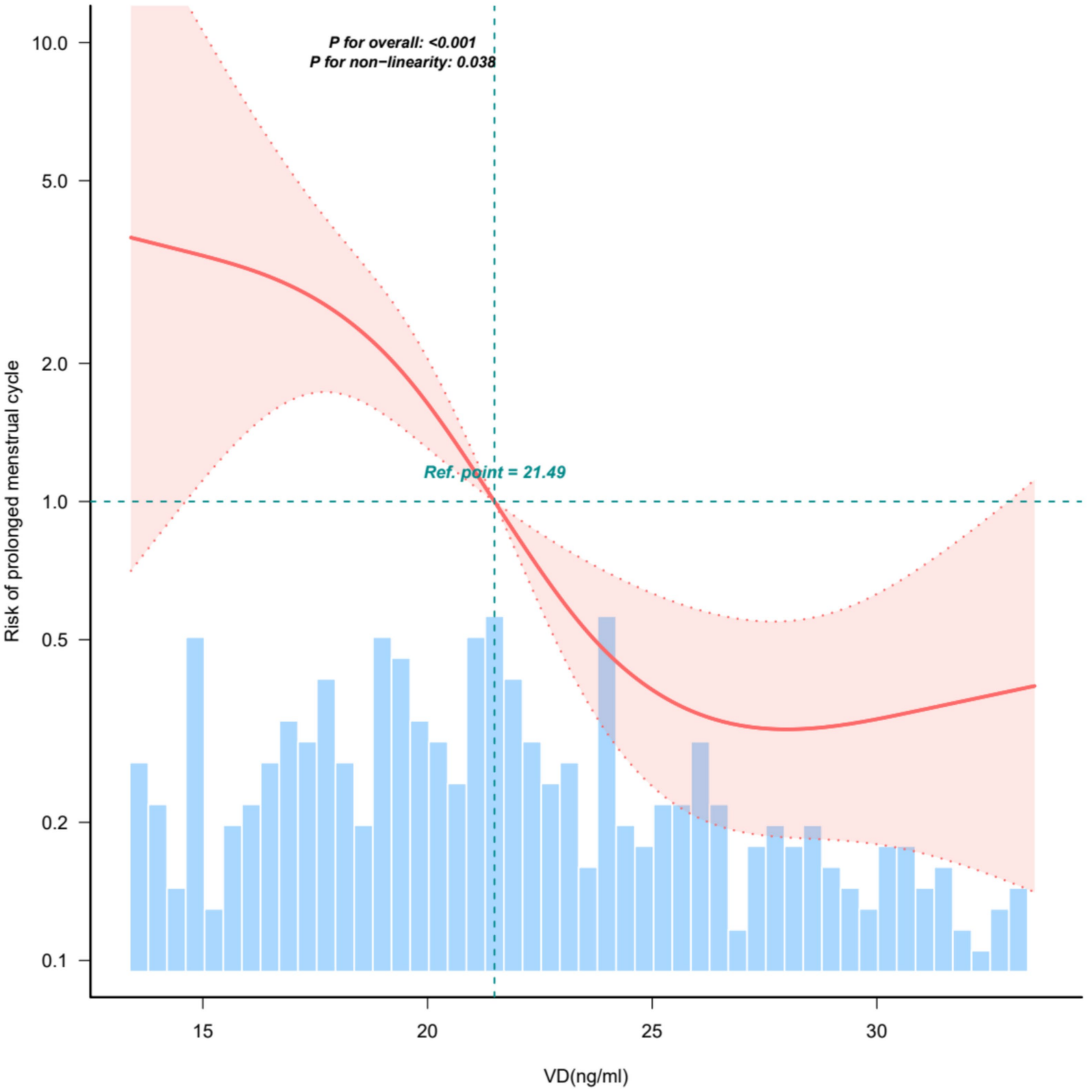
Curve fitting. The non-linearity relationship between vitamin D levels and prolonged menstrual cycle (*p* = 0.038). Figure showed data between the 5th and 95th percentiles. The shading portion of the image indicated the 95% confidence intervals. The odds ratio of the horizontal dashed line was 1.0 (reference point). The reference point was the median VD (21.49 ng/mL). Model was adjusted for age, BMI, HOMA-IR, TT.

The curve fitting showed a non-linear relationship between 25(OH)D and prolonged menstrual cycle risk (*p* = 0.038) (Figure 1). Inflection Point Analysis, adjusted for age, BMI, HOMA-IR, and TT, revealed an inflection point at 27.76 ng/mL (95% CI: 27.41–28.10) (Table 3). Below this threshold, increasing 25(OH)D concentrations were associated with a significant reduction in the risk of prolonged menstrual cycles (Slope 1: OR = 0.82, 95% CI: 0.75–0.89, *p* < 0.001). This indicates that for every 1 ng/mL increase in VD, the risk of prolonged menstrual cycles decreases by approximately 18%. Above 27.76 ng/mL, no significant association was observed (Slope 2: OR = 1.38, 95% CI: 0.97–1.96; *p* = 0.0758). This suggests that once VD exceeds 27.76 ng/mL, menstrual cycle prolongation risk remains stable regardless of additional VD increases. ROC curve analysis demonstrated that at a serum vitamin D cut-off value of 27.76 ng/mL, the sensitivity was 63.48%, specificity was 75.96%, and accuracy was 66.37% [area under the curve (AUC)] (95% CI): 0.695 (0.636–0.754) (Supplementary Figure S1).

**Table 3:** Inflection point analysis.

Item	Breakpoint.OR (95%CI)	*P* value
E_BK1	27.76 (27.41, 28.10)	NA
Slope 1	0.82 (0.75 ~ 0.89)	<0.001
Slope 2	1.38 (0.97 ~ 1.96)	0.0758
Likelihood ratio test	–	0.002
Non-linear Test*1	–	0.032
Non-linear Test*2	–	0.038

The non-linearity relationship between vitamin D levels and prolonged menstrual cycle. Data points represent values within the 5th to 95th percentile range (90% coverage). Model was adjusted for age, BMI, HOMA-IR, TT.

### Subgroup analysis

Subgroup analyses assessed the association between 25(OH)D levels and risk of prolonged menstrual cycles across age, BMI, and LH/FSH ratio subgroups (Table 4), with adjustments for age, BMI, HOMA-IR, and TT. Among women aged <30 years (*n* = 238), higher 25(OH)D concentrations were significantly associated with reduced risk of prolonged menstrual cycles (adjusted OR = 0.87, 95%CI: 0.82–0.93; *p* < 0.001). No significant association was observed in those ≥30 years (adjusted OR = 0.98, 95% CI: 0.92–1.05; *p* = 0.536). A significant interaction was detected between age strata (P-interaction = 0.012), suggesting effect modification by age, with a more pronounced beneficial association in younger PCOS women. No significant interactions were observed for BMI (P-interaction = 0.194) or LH/FSH ratio subgroups (P-interaction = 0.23).

**Table 4:** Subgroup analysis.

Subgroup	Variable	n.total	n.event_%	crude.OR_95CI	crude.P_value	adj.OR_95CI	adj.P_value	P.for.interaction
Age<30	VD	238	184 (77.3)	0.89 (0.85 ~ 0.94)	<0.001	0.87 (0.82 ~ 0.93)	<0.001	0.012
Age≥30	VD	211	161 (76.3)	0.92 (0.87 ~ 0.97)	0.002	0.98 (0.92 ~ 1.05)	0.536	
BMI < 24	VD	283	210 (74.2)	0.92 (0.88 ~ 0.96)	<0.001	0.93 (0.88 ~ 0.97)	0.003	0.194
BMI ≥ 24	VD	154	126 (81.8)	0.87 (0.8 ~ 0.94)	<0.001	0.86 (0.78 ~ 0.96)	0.006	
LH/FSH < 1	VD	172	125 (72.7)	0.90 (0.84 ~ 0.95)	<0.001	0.89 (0.83 ~ 0.95)	0.001	0.230
LH/FSH ≥ 1	VD	194	158 (81.4)	0.94 (0.89 ~ 0.99)	0.020	0.93 (0.88 ~ 0.99)	0.020	

Adjusted: age+BMI+HOMA-IR+TT.

## Discussion

This cross-sectional study, involving 449 patients diagnosed with PCOS, provided an analysis of the relationship between serum 25(OH)D levels and the risk of prolonged menstrual cycles. Our key findings demonstrate that after adjusting for potential confounders including age, BMI, HOMA-IR, and TT, lower serum 25(OH)D levels were independently associated with a significantly increased risk of prolonged menstrual cycles. A critical inflection point for serum 25(OH)D levels was identified at approximately 28 ng/mL. When serum 25(OH)D levels fell below this threshold, the risk of prolonged menstrual cycles decreased significantly as levels increased; beyond 28 ng/mL, the risk plateaued, showing no significant further changes.

This study confirmed that serum 25(OH)D level was a significant factor associated with prolonged menstrual cycles in PCOS patients. Specifically, the rate of prolonged menstrual cycles was significantly higher in the low VD group compared to the normal VD group. This association remained robust after further adjustment for key confounders such as age, BMI, HOMA-IR, and TT. This indicated that lower serum 25(OH)D levels were an independent predictor for prolonged menstrual cycles, independent of common metabolic and endocrine disturbances in PCOS, such as obesity, insulin resistance, and hyperandrogenemia. These findings are consistent with previous studies, suggesting that vitamin D plays a significant role in PCOS-related reproductive dysfunction (11, 21). The underlying mechanism may involve the wide distribution of the vitamin D receptor (VDR) in hypothalamic, pituitary, and ovarian tissues (4, 5, 22, 23). Vitamin D and its receptors may participate in the pathogenesis of PCOS by modulating luteinizing hormone levels, sex hormone-binding globulin concentrations, and testosterone (24). Supporting evidence from animal studies includes observations of delayed ovarian development in vitamin D3-deficient diet-fed mice under controlled conditions (25). It should be noted that our cross-sectional analysis did not detect significant differences in baseline reproductive hormone concentrations across vitamin D subgroups. The manifestation of this phenomenon may be attributable to the following factors: limited sample size for detecting subtle differences, pulsatile nature of reproductive hormone secretion or vitamin D’s likely role in supporting homeostatic regulation rather than acutely modulating baseline levels. The precise mechanisms warrant further investigation.

The most novel finding of this study was the identification of a serum 25(OH)D level of 28 ng/mL as a critical threshold for prolonged menstrual cycle risk. This observation holds substantial clinical relevance. First, 30 ng/mL is a cut-off for distinguishing vitamin D sufficiency from insufficiency, according to classifications that include deficiency, insufficiency, and sufficiency (16). The 28 ng/mL threshold exceeds the internationally accepted upper limit for vitamin D deficiency (typically 20 ng/mL) (16), suggesting that achieving a “non-deficient” status (>20 ng/mL) may be insufficient for maintaining regular cycles in PCOS. An optimal level (>28 ng/mL) appears necessary to reduce oligomenorrhea risk. Second, current vitamin D supplementation guidelines, primarily targeting skeletal health or general populations (16), may require refinement. Most studies on bone health indicate that 20 ng/mL serves as a critical threshold for skeletal integrity (26), whereas for other health outcomes—such as metabolic and reproductive functions—this threshold may be different and often higher. For PCOS-related menstrual irregularity interventions, higher target concentrations (e.g., ≥30 ng/mL) are likely needed to consistently exceed the 28 ng/mL threshold for reproductive benefit. These findings underscore the necessity of establishing outcome-specific vitamin D targets. While these findings highlight a potentially critical threshold and its clinical implications, it is important to acknowledge that the cross-sectional design of this study cannot establish causality. Therefore, the optimal threshold identified requires further validation through interventional studies.

The potential mechanism underlying the vitamin D threshold phenomenon is intrinsically linked to its biological roles in the reproductive system and receptor dynamics. Previous studies have demonstrated that the biosynthetic and signaling systems of vitamin D are expressed in the ovarian follicles of primates, suggesting that vitamin D3 (VD3) modulates follicular development in a stage-dependent manner and may exert direct physiological effects locally within the ovary (27). However, the biological effects of VD3 are mediated through its receptor (VDR). In the presence of ligands, VDR forms a heterodimeric complex with the retinoid X receptor (RXR), denoted as VDR/RXR (28). This complex regulates downstream gene expression by binding to vitamin D response elements (VDREs) in the promoter regions of target genes, thereby facilitating its biological effects (29). Notably, human VDR is ubiquitously distributed across various tissues and organs (30). Animal studies further reveal that VD3 concentration dynamically modulates the quantity of VDR/RXR heterodimers at binding sites, thereby influencing VDR expression levels (31, 32). Based on these findings, the “receptor saturation” theory posits that once serum 25(OH)D concentration reaches a specific threshold, VDR activation approaches a state of saturation. Beyond this point, further increases in vitamin D concentration fail to significantly enhance its biological efficacy, thereby elucidating the observed risk plateau phenomenon (33, 34). Other studies have also indicated that VD3 exhibits non-genomic effects and partially inhibits NMDA and kainate receptor-mediated actions on GnRH neurons, suggesting that VitaD3 may play a regulatory role in the hypothalamic–pituitary-gonadal (HPG) axis during pubertal development (35).

Subgroup analyses revealed a more robust inverse association between vitamin D status and prolonged menstrual cycle risk among PCOS patients aged <30 years. Crucially, baseline vitamin D concentrations in this younger cohort were significantly lower than those in older participants, potentially contributing to the amplified association observed. The elevated prevalence of vitamin D deficiency in younger populations may reflect modifiable lifestyle factors, notably diminished outdoor activity and routine sun-protective measures (36). Notably, fitted curve analysis demonstrated that this inverse correlation remained statistically significant only at vitamin D concentrations below 28 ng/mL. Consequently, whether age independently modifies this association remains indeterminate, necessitating validation through prospective cohort studies.

This study has several limitations. First, the cross-sectional design precludes establishing causal relationships between vitamin D levels and prolonged menstrual cycles. Second, menstrual cycle assessment relied on self-reporting, which is susceptible to recall bias. Future studies should incorporate digital tracking tools to improve accuracy. Third, although adjustments were made for key confounders (age, BMI, HOMA-IR, TT), the study omitted systematic collection of data concerning intergenerational behavioral patterns—specifically sun exposure habits, dietary supplement usage patterns, and outdoor activity duration—or contemporaneous indicators of cultural shifts. Fourth, the analysis entailed missing data for selected covariates. To mitigate this limitation, three complementary methodological approaches were employed: complete case analysis (listwise deletion), single imputation, and multiple imputation. Critically, the primary associations demonstrated consistent effect estimates across all analytical methods, underscoring the resilience of our findings to data gaps. Fifth, the participants were exclusively recruited from a single tertiary reproductive medicine clinic; consequently, the sample may represent a PCOS population with more severe clinical manifestations. Therefore, the generalizability of these findings to community-based or non-Chinese populations requires cautious interpretation. Collectively, these limitations underscore the need for prospective cohort studies to dynamically evaluate temporal associations between vitamin D status changes and menstrual cycle improvements, thereby clarifying causality and clinical utility.

## Conclusion

Following adjustment for key confounders, this cross-sectional study revealed an independent association between vitamin D deficiency and an increased likelihood of prolonged menstrual cycles in patients with PCOS. An exploratory threshold of 28 ng/mL for serum 25(OH)D was derived from non-linear modeling. Below this level, an inverse dose–response pattern was observed, wherein higher vitamin D concentrations correlated with progressively lower odds of cycle prolongation. Beyond this threshold, the association appeared to stabilize. A more pronounced inverse association was observed in patients below 30 years of age, where a higher prevalence of vitamin D deficiency was also found. Collectively, these findings suggest a potential nonlinear relationship between vitamin D status and menstrual cycle regularity in PCOS. They provide preliminary epidemiological support for the hypothesis that optimizing vitamin D status might be relevant to improving menstrual cyclicity, which merits validation in longitudinal and interventional studies.

## Data Availability

The raw data supporting the conclusions of this article will be made available by the authors, without undue reservation.
