# Correction: Vitamin D levels and prolonged menstrual cycle in women with polycystic ovary syndrome: a cross-sectional study

**DOI:** 10.3389/fnut.2026.1885084

**Published:** 2026-06-23

**Authors:** Lijing Wang

**Affiliations:** Department of Reproductive Health and Infertility, Zhaoqing Maternal and Child Health Care Hospital, Zhaoqing, China

**Keywords:** fitted curve, inflection point analysis, PCOS, prolonged menstrual cycle, vitamin D

There were some errors in Table 2 as published. Upon reexamination with the guidance of a professional statistician, we found that the Model Nagelkerke *R*^2^ values for the various models were incorrect. These have now been corrected, while all other statistical results remain unchanged. The corrected Table 2 appears below.

**Table 2:** Logistic multivariate analysis of vitamin D levels and prolonged menstrual cycle.

Model No.	Variable	OR (95%CI)	*P*	Model Nagelkerke *R*^2^	Model significance *P*
Model 1	VD	0.90 (0.87 ~ 0.94)	< 0.001	0.110	< 0.001
Model 2	VD	0.91 (0.87 ~ 0.94)	< 0.001	0.114	< 0.001
	Age	1.00 (0.95 ~ 1.05)	0.857		
	BMI	1.05 (0.99 ~ 1.11)	0.078		
Model 3	VD	0.91 (0.87 ~ 0.94)	< 0.001	0.129	< 0.001
	Age	1.00 (0.95 ~ 1.05)	0.857		
	BMI	1.05 (0.99 ~ 1.11)	0.078		
	HOMA	1.21 (1.07 ~ 1.36)	0.002		
	TT	2.13 (0.71 ~ 6.42)	0.177		

Model 1: crude model.

Model 2: adjusted for age+BMI.

Model 3: adjusted for age+BMI+HOMA-IR+TT.

The original version of this article has been updated.

