# Supplementary material for: Vitamin D levels and prolonged menstrual cycle in women with polycystic ovary syndrome: a cross-sectional study
Source: Front Nutr. 2026 May 13;13:1785886. doi: 10.3389/fnut.2026.1785886 (PMC13212045; doi:10.3389/fnut.2026.1785886)
Supplement: Supplementary file 1 [file Image_1.pdf]

Supplementary Figure S1. ROC Curue

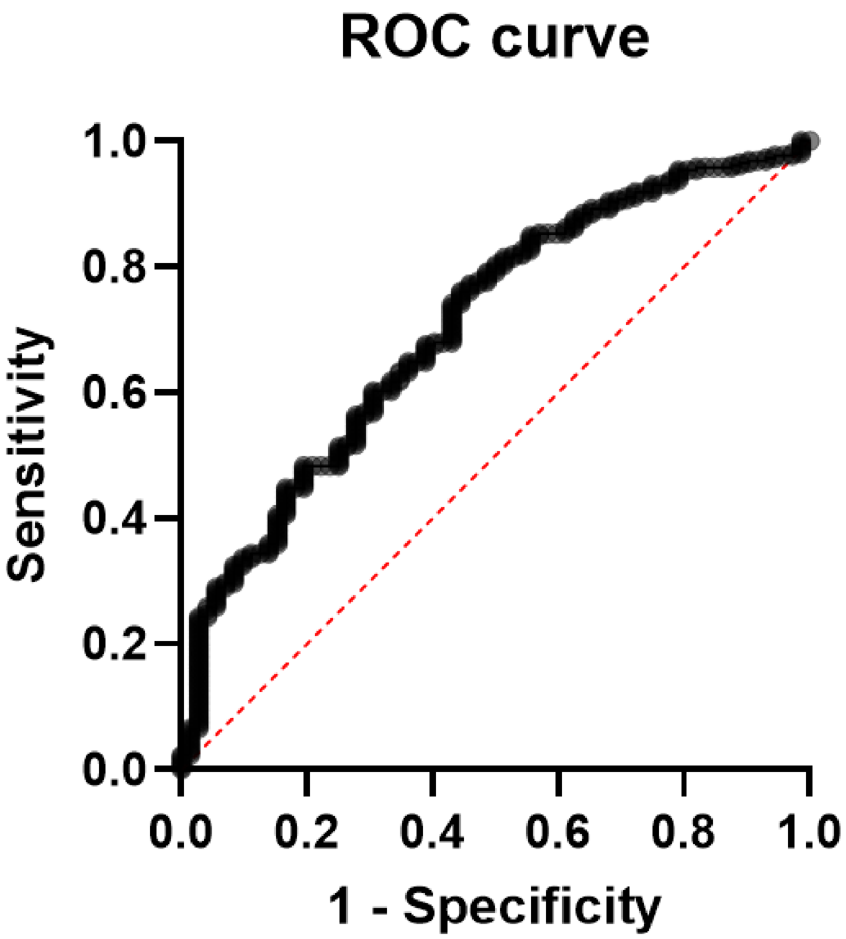

Supplementary Figure S1. ROC curve analysis for sensitivity and specificity of serum 25(OH)D cutoff value of 27.76 ng/ml for predicting the risk of prolonged menstrual cycle.
