# Supplementary material for: Vitamin D levels and prolonged menstrual cycle in women with polycystic ovary syndrome: a cross-sectional study
Source: Front Nutr. 2026 May 13;13:1785886. doi: 10.3389/fnut.2026.1785886 (PMC13212045; doi:10.3389/fnut.2026.1785886)
Supplement: Supplementary file 2 [file Table_1.DOCX]

Supplementary Table S1. Missing values for the covariates

| Variables | Missing value | Missing value ratio (%) |
| --- | --- | --- |
| VD（ng/ml） | 0 | 0 |
| Menstrual cycle | 0 | 0 |
| Age (y) | 0 | 0 |
| BMI (Kg/m^2^) | 12 | 2.67 |
| TSH(uIU/ml) | 70 | 15.59 |
| HCY(umol/L) | 54 | 12.03 |
| FPG (mmol/L) | 35 | 7.80 |
| FINS (mU/L) | 40 | 8.91 |
| HOMA-IR | 40 | 8.91 |
| A4(nmol/L) | 105 | 23.39 |
| SHBG(nmol/L) | 105 | 23.39 |
| FSH(IU/L) | 80 | 17.81 |
| LH(IU/L) | 80 | 17.81 |
| TT(ng/ml) | 80 | 17.81 |
| E2(pg/ml) | 80 | 17.81 |
| P4(ng/ml) | 80 | 17.81 |
| PRL(ng/ml) | 80 | 17.81 |
| LH/FSH | 80 | 17.81 |

BMI, Body mass index; TSH, Thyroid stimulating hormone; HCY, Homocysteine; FPG, Fasting plasma glucose; FINS, Fasting insulin; HOMA-IR, Homeostatic Model Assessment of Insulin Resistance; A4, Androstenedione; SHBG, Sex hormone binding globulin; FSH, Follicle stimulating hormone; LH, Luteinizing hormone; E2, Estradiol; TT, Total testosterone; P4, Progesterone; PRL, Prolactin.
