# Supplementary material for: Vitamin D levels and prolonged menstrual cycle in women with polycystic ovary syndrome: a cross-sectional study
Source: Front Nutr. 2026 May 13;13:1785886. doi: 10.3389/fnut.2026.1785886 (PMC13212045; doi:10.3389/fnut.2026.1785886)
Supplement: Supplementary file 3 [file Table_2.DOCX]

Supplementary Table S2. Variance Inflation Factor Test

| Term1 | coeff1 | Change.  percentage1 | Term2 | coeff2 | Change.  percentage2 | VIF |
| --- | --- | --- | --- | --- | --- | --- |
| Crude | -0.10 | Ref. | Full | -0.09 | Ref. | 1.030 |
| age | -0.10 | 0.7 | age | -0.08 | -4.6 | 1.140 |
| BMI | -0.10 | -2 | BMI | -0.09 | -2.6 | 1.686 |
| HOMA | -0.09 | -7.7 | HOMA | -0.09 | 4.6 | 1.684 |
| TT | -0.09 | -12 | bT | -0.09 | 6.6 | 1.138 |

Multicollinearity among covariates was quantitatively examined using the Variance Inflation Factor (VIF) test.
