# Supplementary material for: Vitamin D levels and prolonged menstrual cycle in women with polycystic ovary syndrome: a cross-sectional study
Source: Front Nutr. 2026 May 13;13:1785886. doi: 10.3389/fnut.2026.1785886 (PMC13212045; doi:10.3389/fnut.2026.1785886)
Supplement: Supplementary file 5 [file Table_4.DOCX]

Supplementary Table S4. Logistic multivariate analysis of vitamin D levels and prolonged menstrual cycle (complete case analysis)

| Variable | n.total | Model 1  OR(95%CI) | P | Model 2  OR(95%CI) | P | Model 3  OR(95%CI) | P |
| --- | --- | --- | --- | --- | --- | --- | --- |
| VD | 335 | 0.92  (0.88~0.96) | <0.001 | 0.91 (0.87~0.94) | <0.001 | 0.92 (0.88~0.96) | <0.001 |

Model 1: crude model

Model 2: adjusted for age+BMI

Model 2: adjusted for age+BMI+HOMA+TT
